# Surface Compositional Change of Iron Oxide Porous Nanorods: A Route for Tuning their Magnetic Properties

**DOI:** 10.3390/molecules25051234

**Published:** 2020-03-09

**Authors:** Alberto Casu, Danilo Loche, Sergio Lentijo-Mozo, Andrea Falqui

**Affiliations:** NABLA Lab, Biological and Environmental Sciences and Engineering (BESE) Division, King Abdullah University of Science and Technology (KAUST), Thuwal 23955–6900, Saudi Arabia; danilo.loche@kaust.edu.sa (D.L.);

**Keywords:** nanorods, cation exchange, magnetic nanoparticles, core/shell, nanopores

## Abstract

The capability of synthesizing specific nanoparticles (NPs) by varying their shape, size and composition in a controlled fashion represents a typical set of engineering tools that tune the NPs magnetic response via their anisotropy. In particular, variations in NP composition mainly affect the magnetocrystalline anisotropy component, while the different magnetic responses of NPs with isotropic (i.e., spherical) or elongated shapes are mainly caused by changes in their shape anisotropy. In this context, we propose a novel route to obtain monodispersed, partially hollow magnetite nanorods (NRs) by colloidal synthesis, in order to exploit their shape anisotropy to increase the related coercivity; we then modify their composition via a cation exchange (CE) approach. The combination of a synthetic and post-synthetic approach on NRs gave rise to dramatic variations in their magnetic features, with the pores causing an initial magnetic hardening that was further enhanced by the post-synthetic introduction of a manganese oxide shell. Indeed, the coupling of the core and shell ferrimagnetic phases led to even harder magnetic NRs.

## 1. Introduction

Iron oxides attract considerable attention due to their many physical properties, which vary greatly among different phases and represent the key to their successful use in a wide array of applications, from hyperthermia, magnetic resonance imaging and drug delivery, to magnetic separation, catalysis and magnetic data storage [1,2,3,4,5,6,7]. Given the richness of the iron/oxygen phase diagram [8], enhancing the performance of any iron oxide phase for such diverse purposes translates into the necessity to master the production of particles of controlled composition, size and shape in order to control the physical features that drive each particular response. When considering the possible applications that revolve around the magnetic response of iron oxides, modifying the magnetic hardness and/or saturation magnetization of the nanoparticles (NPs) up to values equal or higher than bulk ones represents one of the biggest existing challenges, since it aims at obtaining nanosized objects capable of equaling or overcoming the current limits of a given material. To reach these goals, different interconnected aspects, such as the structural order and the shape of the NPs, become fundamental in determining the final features of magnetic NPs. In fact, the choice of an anisotropic shape over the spherical isotropic shape increases the shape component of the effective magnetic anisotropy constant, while any variation in the NP composition affects the magnetocrystalline anisotropy, and both have an impact on the net magnetic hardness. Still, both kinds of modification can be dramatically affected by the loss of structural order connected with the lattice distortions introduced by the faceting required to obtain any given shape (the so-called superficial dead layer) or by the introduction of different atoms or phases in the ideal, bulk-like matrix. The superficial or structural disorder manifests as the difficulty or even impossibility to obtain a full alignment of a portion of the atomic magnetic moments, which then provide a lowered or null contribution to the net magnetization of the NPs [9]. Starting from this general situation, we propose a novel and atypical two-step approach to boost the magnetic hardness of elongated NPs of magnetite, i.e., nanorods (NRs). First, we synthesized partially hollow magnetite (Fe_3_O_4_) NRs by reducing nanospindles of akageneite (β-FeOOH) and used the presence of internal voids (nanopores) to obtain a sort of core/double shell-like system, with the double shell consisting of the internal (i.e., belonging to the pores) and external structurally disordered surfaces of the NRs, which commanded a magnetic hardening of the NRs. Then, we used a post-synthetic cation exchange (CE) approach to grow a hausmannite (Mn_3_O_4_) external shell that both cured the structural disorder just on the external surface of the magnetite NRs and magnetically coupled with them, thus giving rise to core/multi-shell nanostructures with a coercivity higher than the one of bulk hausmannite.

## 2. Results

Our approach to obtain mixed Mn_x_Fe_3−x_O_4_ nanorods (NRs) was developed starting from our previous results [10]. The approach consists of a multi-step strategy, described in more detail in the Materials and Methods section, which originates from the studies of Chen M. et al. and Peng et al. [11,12]. The former successfully synthesized acicular β-FeOOH (akaganeite) NPs by hydrolysis of FeCl_3_·6H_2_O, while the latter succeeded in retaining the elongated shape of the NPs during the reduction from β-FeOOH to magnetite using a two-step process. Since then, this approach has been widely adopted and the introduction of polyethylenimine (PEI) polymers has been suggested as a possible stabilizer to finely tune the aspect ratio (AR) of the final NPs [13]. However, we showed that the molecular weight (M_W_) of PEI is a key parameter that modifies the shape of the NPs during both their synthesis and reduction. In fact, by comparing NPs obtained with and without PEI addition, we observed that the introduction of PEI determines a consistent variation in the shape and size of the resulting β-FeOOH NPs and, moreover, the use of PEI polymers with different M_W_ also gave rise to dramatic variations in the shape and size of the final products obtained by reduction of β-FeOOH NRs [10].

**Figure 1:**
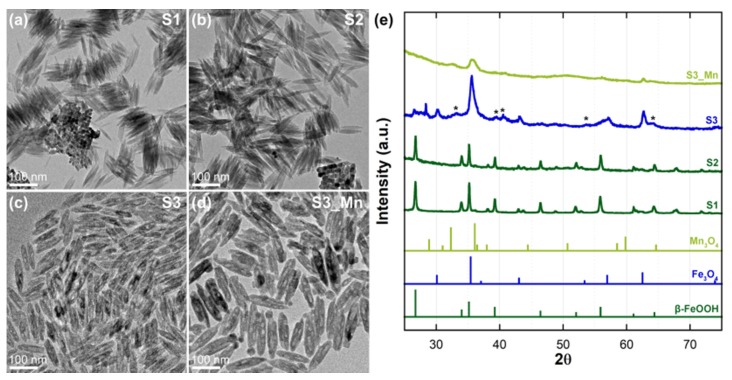
Conventional TEM imaging of the samples: (**a**) S1; (**b**) S2; (**c**) S3; (**d**) S3_Mn. Panel (**e**) reports the XRD pattern of all samples. The * symbol in the S3 sample’s XRD pattern indicates the peaks coming from hematite residuals.

Thus, we modified our synthetic strategy by combining the initial β-FeOOH spindle-like NPs (sample S1, Figure 1a) with KCl to obtain β-FeOOH(Cl^−^) NRs (sample S2, Figure 1b), which maintained the nanospindles’ elongated morphology and improved their stability during reduction, with minimal variations in terms of size (Table 1). In fact, as already showed by Wu et al. in their study on hematite nanostructures [14], the introduction of inorganic salts provides additional binding sites at the surface of the NPs that increase the thermal stability of the NPs. This variation manifests as a dampening effect on the fast weight loss usually expected during reduction of β-FeOOH, and helps in preserving the NPs’ morphology, as also highlighted by the vertically self-assembled nanospindles of Figure 1a,b which clearly show their square-shaped cross section. This assumption was proven right with the reduction of S2 with oleylamine (OLA): contrarily to what we previously observed during the reduction of nanospindles without KCl [10], the reduction of nanospindles already subject to KCl gave rise to new, shorter NRs (sample S3, Figure 1c) that maintained the high aspect ratio (AR) of the previous synthetic steps. The most visible morphological variations observed after reduction were not only the rounding of the nanospindles’ tips (thus the NPs in S3 are indicated as NRs rather than nanospindles) but also the pervasive formation of nanopores along the whole NPs length. The final step, represented by the introduction of Mn by a cation exchange (CE) protocol (sample S3_Mn, Figure 1d) also did not affect the morphology of the final NPs (sample S3_Mn), with slight increases in their mean length and width (Table 1).

The achievement of each synthetic step was initially monitored by XRD and the corresponding XRD patterns are reported in Figure 1e. The peaks observed in diffraction patterns of S1 and S2 are consistent with the presence of β-FeOOH (PDF card 34–1266) and confirm our previous findings [10], while those relative to S3 indicate the formation of a magnetite (PDF card 19–629) main phase along with the possible presence of residuals, such as hematite (α-Fe_2_O_3_, peaks indicated by the * symbol, PDF card 33–0664). Finally, the peaks observed in the diffraction pattern of S3_Mn are compatible with the presence of magnetite along with manganese oxide (Hausmannite, Mn_3_O_4_, PDF card 24–734).

**Figure 2:**
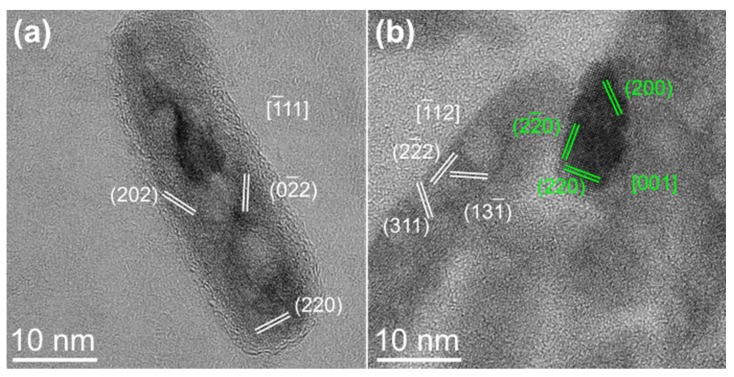
HRTEM imaging of: (**a**) S3 and (**b**) S3_Mn. Magnetite lattice planes are indicated in white, while those of hausmannite (Mn_3_O_4_) are indicated in green.

Since the main focus of our studies revolves around the structural and magnetic characteristics of the ferrimagnetic (FiM) phases of our final samples S3 and S3_Mn, we further observed their structural evolution (i.e., the appearance of nanopores and the formation of different phases within the NRs) by HRTEM to gain an additional local insight on their finer characteristics (Figure 2). In both cases the NRs showed single crystal features, indicating that the formation of nanopores during reduction and the introduction of Mn during CE did not affect the NRs radically from a structural point of view and did not determine their transition from single crystals to polycrystals. In more detail, the structural analysis of the NRs confirmed the presence of magnetite in S3, while the manganese oxide previously indicated by XRD was observed mainly in the external regions of the S3_Mn NRs, suggesting a core/shell configuration with single crystals of iron oxide as cores and Mn-rich oxides as shells, with occasionally thicker, irregular Mn oxide lumps.

**Figure 3:**
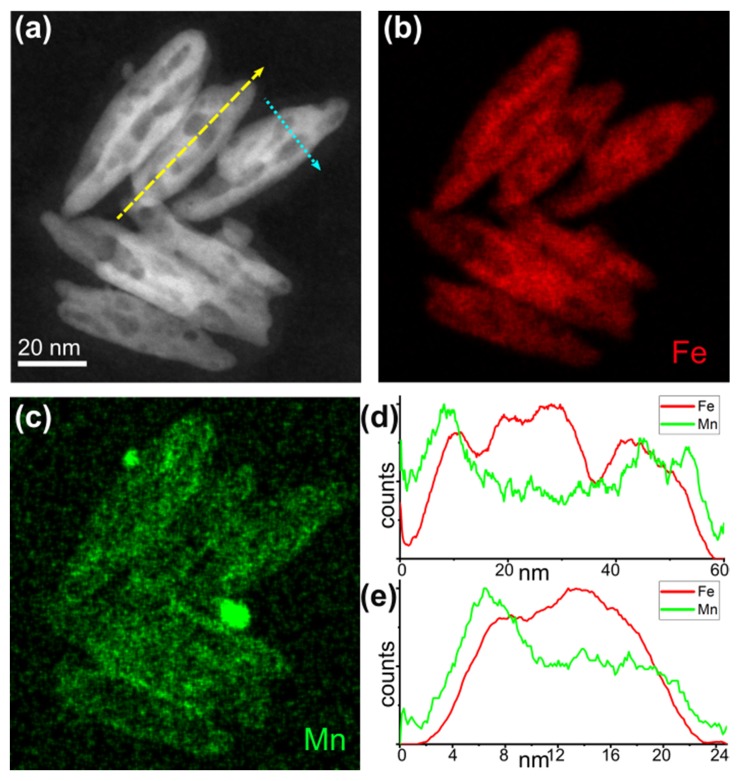
Sample S3_Mn. (**a**) HAADF-STEM image, (**b**) Fe map (red), (**c**) Mn map (green); (**d**) compositional profile of Fe and Mn along the length of the NR (yellow dashed line of panel (**a**)), (**e**) compositional profile of Fe and Mn along the width of the NR (cyan dotted line of panel (**a**)).

This assumption was further corroborated by elemental mapping of the S3_Mn NRs performed by Energy-Dispersive X-ray Spectroscopy performed by Scanning Transmission Electron Microscopy in High Angular Annular Dark Field geometry (HAADF-STEM EDS) (Figure 3), which highlighted the formation of an irregular Mn-rich shell around the Fe-rich starting NRs, which became the cores of core/shell nanostructures with a manganese content of 9%. Such a result is additionally confirmed by the compositional profiles measured along the length and width of the NRs, which show that Fe is present along the NRs but only Mn is present at their extremities. In fact, the Fe signal along the compositional profiles is dome-shaped with small drops in correspondence with the nanopores, as expected for a core, while the Mn signal follows the general M-shaped profile expected for a shell.

**Figure 4:**
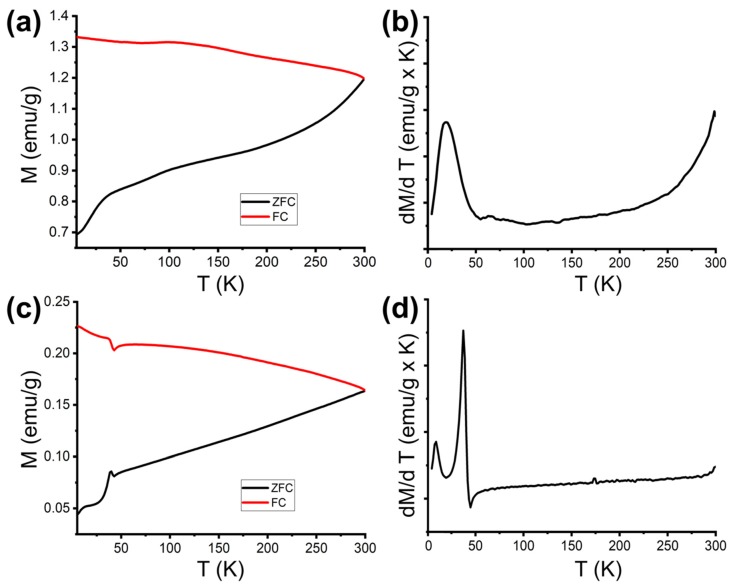
Zero-field-cooled/field-cooled (ZFC-FC) magnetization curves and their derivative of samples S3 and S3_Mn: (**a**) ZFC-FC curves of S3, (**b**) derivative curve (d(M_ZFC_–M_FC_)/dT vs. T) of sample S3, (**c**) ZFC-FC curves of sample S3_Mn; (**d**) derivative curve (d(M_ZFC_–M_FC_)/dT vs. T) of sample S3_Mn.

S3 and S3_Mn were also investigated by DC magnetometry to study the effect of a high AR, anisotropic shape on the magnetic features of single crystals of iron oxide (S3) and core/shell systems (S3_Mn). Both samples are still magnetically blocked at room temperature, as shown by Zero-field-cooled/field-cooled (ZFC-FC) curves (Figure 4), which do not display T_B_ and T_IRR_ within the experimental range (Table 2). The sharp peak in magnetization observed at 40 K in the ZFC (zero-field-cooled) and FC (field-cooled) curves of S3_Mn and the corresponding spike observed in the derivative curve (T_MAX_) are consistent with the transition of Mn_3_O_4_ from a ferrimagnetic to a paramagnetic (FiM-to-PM) state, while the first peak observed in the derivative curve of S3 can be attributed to the reorientation and subsequent unblocking of the disordered magnetic domains constituting the so-called dead layer at the surface of the NRs [15,16].

**Figure 5:**
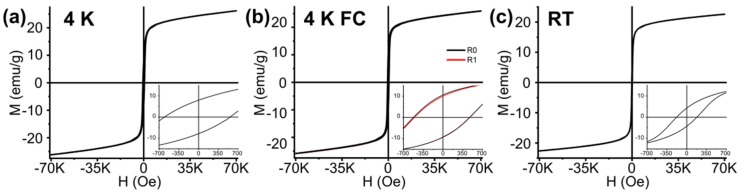
Hysteresis loops of sample S3 with maximum applied field equal to 70 kOe. The insets show the low field regions of each loop. (**a**) Hysteresis recorded at 4 K; (**b**) Hysteresis recorded at 4 K after cooling under an applied field equal to 70 kOe. R0 indicates the hysteresis recorded after cooling, R1 refers to the hysteresis recorded immediately after the first one; (**c**) Hysteresis recorded at RT.

**Figure 6:**
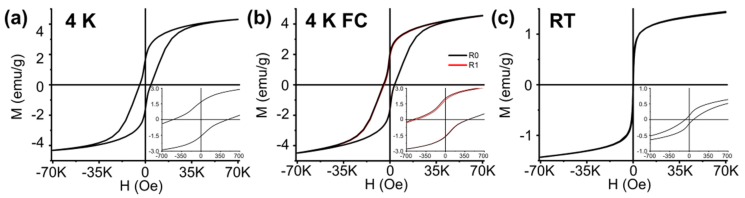
Hysteresis loops of sample S3_Mn with maximum applied field equal to 70 kOe. The insets show the low field regions of each loop. (**a**) Hysteresis recorded at 4 K; (**b**) Hysteresis recorded at 4 K after cooling under an applied field equal to 70 kOe. R0 indicates the hysteresis recorded after cooling, R1 refers to the hysteresis recorded immediately after the first one; (**c**) Hysteresis recorded at RT.

The zero field cooled hysteresis loops recorded at 4 K show the features of S3 and S3_Mn in their magnetically blocked state: both samples are characterized by low values of saturation magnetization (M_S_) when compared to their bulk counterparts (M_S_ values of Fe_3_O_4_ and Mn_3_O_4_ being 92 emu/g, and 38 emu/g, respectively) and by widely different coercivity values, namely 581 Oe for S3 and 4201 Oe for S3_Mn (Figure 5a, Figure 6a, Table 3). Furthermore, both hysteresis loops display a non-null exchange bias (i.e., horizontal shift of the hysteresis), which becomes apparent in the FC hysteresis loops recorded at 4 K (Figure 5b, Figure 6b), where R0 and R1 indicate two consecutive hysteresis loops recorded to highlight possible training effects. Hysteresis loops recorded at RT (room temperature, 300 °C) do not display any shift, but it is worth pointing out a remarkable variation in coercivity values, i.e., 179 Oe for S3 and 74 Oe for S3_Mn (Figure 5c, Figure 6c, Table 3).

## 3. Discussion

Our synthetic approach allowed us to obtain akaganeite NRs with a 6.6 AR (aspect ratio) and an average length of 99.2 nm, which is an intermediate size between that of the nanospindles obtained without PEI and that of NRs obtained using PEI with a different molecular weight [10]. These features were mostly maintained after the introduction of KCl, with a slightly lower AR and length (now 6.0 and 96.5 nm, respectively) paired with an improved monodispersity of the S2 NRs, as indicated by the lower standard deviation values in both length and width (standard variation for length and width being 16.8 nm and 2.7 nm for S1 and 9.8 nm and 1.6 nm for S2, respectively). The S2 NRs maintained their shape during reduction with OLA in N_2_ atmosphere, albeit with an overall shortening (AR of the S3 NRs is 4.2, average length is 60.7 nm). This outcome is similar to what was previously observed by Wu et al. [14] in their study to obtain hematite NRs by calcination of FeOOH NRs, but in our case the shortening is much more limited and the thinning is minimal, suggesting that the effect of reduction on the starting NPs was less destructive. The reduction, however, also determined the formation of pores within the nanorods. Similar phenomena had been already observed by Wu et al. during the decomposition of akaganeite NRs to hematite [14] and by Jazinehpour et al. during the reduction of goethite NPs to magnetite [17]. In both cases the pores were ascribed to the rearrangement of the NPs during the process. In particular, according to Wu et al. the formation of pores is due to tunnels that are typical of the akageneite structure and that make it prone to take in Cl anions. These anions act as dampers during the reduction, thus helping to maintain the elongated shape of the NRs during the process; however, their release also triggers the formation of pores, as shown by TEM and HRTEM in Figure 1 and Figure 2. The increased size of the S3_Mn NRs following the introduction of Mn can be explained in the light of the compositional analysis by EDS as the effect of the formation of a Mn-rich shell on top of the S3 NRs. This result is in good accordance with previous studies conducted by our group [10,18] and once again confirms that the superficial disorder is the key parameter driving the initial penetration of Mn ions in the NRs. However, the magnetite structure is not prone to further continue with the CE once the superficial vacancies have been occupied [19].

In this regard, the possible presence of “open” pores in the NRs should in principle foster an increased content of Mn due to the increased surface made available, and to the thinner internal NR volume in the zones near them, which might in turn promote a higher local defectivity and, consequently, an easier path for the deeper penetration of the CE front towards the inner portion of the NRs. The resulting NRs would consequently be inhomogeneous in terms of Mn content, with Mn-richer zones forming in correspondence with the open pores and determining an overall “patched” distribution of Mn in the elemental maps. Conversely, the distribution of Fe and Mn in the elemental maps shown in Figure 3 follows the variations in brightness of the HAADF-STEM images. Thus, given the similarity between Mn and Fe in terms of Z-contrast, this means that the brighter zones correspond mainly to thicker ones. Furthermore, no major variation in Mn content can be observed in correspondence with the pores. These results suggest that the nanopores formed during the reduction of NRs are indeed internal and completely isolated from the external surface, hence they do not act as a second CE front for Mn.

The magnetic behavior of S3 and S3_Mn (the reduced NRs pre- and post-CE with Mn) was also studied to verify how the reduction process and the introduction of Mn affect the NR properties. At first, the DC magnetization was studied as a function of temperature upon low field condition (50 Oe) according to the ZFC-FC protocols. As mentioned above, in both cases the samples remained in a magnetically blocked state for the whole temperature range (4 ÷ 300 K), but a variation in the slope of both curves can be observed in the low-temperature region, along with a further brisk variation in the ZFC and FC curves of S3_Mn at 42 K, while there is no evidence of Verwey transition around 125 K [20]. The former likely indicates the unfreezing and realigning of small-sized magnetic domains commonly identified as a superficial dead layer, while the latter falls around the ferrimagnetic-to-paramagnetic (FiM-to-PM) transition temperature of Mn_3_O_4_, which takes place at T_C_ = 42 K in both bulk and nanosized manganese oxide [21]. This means that in the low field regime below the transition temperature, the most external layer of FiM Mn_3_O_4_ is ferromagnetically coupled with the core magnetite NRs, while above that temperature only the magnetite and the thin layer partially doped with the Mn (i.e., of likely Mn ferrite) are responsible for the magnetization. The impossibility of observing magnetic phase transitions, such as the Verwey transition, is not uncommon for nanoparticles and is usually attributed to lattice distortions that affect the crystallinity and the magnetic response [22,23], which in our case are likely originated by the internal pores. Thus, while the structural disorder has a smaller effect on the Mn_3_O_4_ shell, which was formed at a later stage, it heavily affects the pristine, porous magnetite phase. In fact, the presence of pores not only modifies the structure of the magnetite NRs, but it also affects their magnetic behavior at different levels, as previously suggested during the study of different iron oxides [22,24]. The pore formation implies a smaller number of atoms with respect to those contained in “full” NRs of similar size, while the presence of additional superficial atoms at the surface of the pores means an additional source of spin canting for the atoms inside the NRs. Both aspects are logically expected to negatively affect the magnetization of the NRs by subtracting atoms capable of fully aligning with the external magnetic field, finally determining lower values of magnetization. This morphological feature provides the logical explanation for the low saturation observed in the ZFC-FC curves and hysteresis loops of S3 when compared with similar NRs and with magnetite spherical NPs [25,26,27,28]. In particular, while the comparison is relatively straightforward when considering the magnetic features of NRs, it is less so for nanospheres. The first point to be considered is the actual dimension of the magnetic domains, which usually corresponds to the NP size in case of single domain nanoparticles. Then, starting from the average width and length of our NRs, their volume can be calculated and corresponds to that of nanospheres around 30 nm in size, which is the maximum size for single domain magnetite NPs [29]. Even assuming this approximation, which does not take into account the presence of hollow pores in the NRs of sample S3 and consequently overestimates the quantity of available Fe atoms to be magnetized in the NRs, the magnetic response of S3 is quite different from that of nanospheres of similar volume [23,28]. In fact, although all the spherical NPs are still magnetically blocked at 300 K, as should be expected given the similar volumes, the NRs constituting the S3 sample show higher coercivity at low temperature and at room temperature, due to their anisotropic shape and to the higher contribution of internal and external superficial disorder. On the other hand, the disorder and the lack of Fe atoms in the zones corresponding to the hollow pores contribute to decreasing the net magnetization, thus driving down the saturation magnetization with respect to the spherical NPs. Furthermore, an even more dramatic effect on the saturation magnetization and coercive field is observed at low temperatures and upon high field regime in S3_Mn, with the former being heavily decreased and the latter being conversely increased in comparison with the corresponding values observed for the S3. Both effects, taking place once Mn has been introduced in the NRs and triggered by the formation of a mixed shell composed of FiM Mn ferrite and FiM manganese oxide, could only be ascribed to an antiferromagnetic (AFM) coupling involving the external shell of manganese oxide, the intermediate shell of Mn ferrite and the porous core of magnetite. Such an AFM coupling further lowers the net saturation magnetization of S3_Mn while making the resulting ensemble of magnetic phases magnetically harder. In fact, while M_S_ becomes almost eight times lower than that measured for the S3 sample, the coercivity measured at 5 K for S3 is roughly five times higher than what was previously observed for similar-sized NRs [25,26] and is even higher than those of much bigger NRs [17]. In this case too, a further comparison can be drawn between the NRs of S3_Mn and spherical NPs of similar volume, but given the multilayered nature of our samples, flower-like isotropic core/shell NPs of magnetite and manganese oxide represent the most fitting counterpart [18]. Both systems are characterized by an AFM coupling between core and shell, but once again the NRs have higher coercivity due to their anisotropic shape, while their saturation magnetization is lowered by the partially hollow magnetite core. Thus, the coercivity of S3_Mn at 4 K, which is higher than the bulk value of Mn_3_O4, should not be only attributed to the presence of a Mn_3_O_4_ FiM shell, but to a joint effect also involving its AFM coupling with the highly disordered, partially hollow FiM core of S3. After the CE reaction, these final nanoparticles act as a composite multi-interface system composed of the disordered inner surfaces of the hollow zones, the main Fe_3_O_4_ core volume and the external interface.

Thus, on one hand the presence and relevance of superficial magnetic disorder is verified by measuring the spontaneous exchange bias (SEB) in the ZFC hysteresis loops recorded in a blocked state. As indicated by their corresponding H_E_ values, S3 and S3_Mn at 4 K are both affected by SEB, which is in fact maximized in S3_Mn. This is in accordance with our previous studies on spherical NPs of different size, where the introduction of a new cation by a CE approach did lower the SEB at first by forming a mixed shell and reducing the initial superficial disorder, but also led to a further increase once the new cation formed a second shell on top of the mixed shell [18,19]. Here, the curing effect of Mn atoms on the superficial disorder of S3, which is limited to the sole external surface, is likely masked by the additional disorder introduced by the newly formed external Mn_3_O_4_ shell. On the other hand, the above-mentioned AFM coupling between the Mn_3_O_4_ FiM shell and the FiM core of S3 becomes further apparent by looking at the conventional exchange bias (CEB) in the FC hysteresis loops recorded at 4 K for both samples. Furthermore, the vertical shift (VS), evidenced by the non-null values of ΔM_R_, and the occurrence of a training effect for repeated hysteresis loops (in both Figure 5 and Figure 6, R0 and R1 are the first and second hysteresis loops) confirm that the superficial disorder of the inner and external surfaces work as makeshift shells from a magnetic point of view, thus suggesting that the superficial magnetic disorder gives rise to spinglass-like magnetic responses [30]. VS is always lower in the FC hysteresis loops of S3_Mn, which proves that the introduction of Mn did also actually have a curing effect on the disorder of the external surface of the magnetite NRs, in agreement with what we recently observed on similar systems [18].

In this framework, the low saturation and high coercivity values of the hysteresis loops recorded at RT confirm the general results obtained at 4 K: the structural disorder given by the pores negatively affects the reorientation of the atoms and considerably lowers the net magnetization, while making the NRs magnetically harder than NRs of similar size [25,26] and comparable with the bulk (H_C_ = 115/150 Oe) [8]. On the other hand, when considering S3_Mn, the Mn_3_O_4_ shell becomes PM above its transition temperature of 42 K and ceases any contribution to the net magnetization or to the magnetic hardness of the sample, while the curing provided by Mn atoms at the magnetite/Mn_3_O_4_ interface decreases the spin canting and lowers the magnetic hardness and the coercivity of the starting S3 sample.

## 4. Materials and Methods

### 4.1. Materials

Oleylamine (OLA, 70%), potassium chloride KCl (99%), the manganese precursor MnCl_2_ (99%) and the iron precursor FeCl_3_ × 6H_2_O (98%) were purchased from Sigma-Aldrich (St. Louis, MO, USA). Tri-octylphospine (TOP, 97%) was purchased from Strem chemicals (Newburyport, MA, USA). Organic solvents like acetone and hexane were of analytical grade and obtained from various sources. All chemicals were used as received without any further purification. Some of the experiments were carried out using standard airless techniques: a vacuum/dry nitrogen gas Schlenk line was used for synthesis and an Ar glovebox for storing and handling air- and moisture-sensitive chemicals.

#### 4.1.1. Synthesis of β-FeOOH Nanorods

A solution of FeCl_3_ × 6H_2_O (20 mmol) in 100 mL of deionized water was heated at 80 °C for 1 h. The as-obtained orange solid precipitate was separated by centrifugation, washed several times with acetone and dried. This sample was labeled S1.

#### 4.1.2. Synthesis of β-FeOOH(Cl^−^) Nanorods

S1 (230 mg, 2.5 mmol) and KCl (7.38 g, 99 mmol) were dissolved in 60 mL of deionized water and heated at 90 °C for 5 h under magnetic stirring. After that, an orange solid was precipitated and separated by centrifugation and washed first with a mixture of H_2_O/acetone (1:3), and then with acetone. This sample was named S2.

#### 4.1.3. Synthesis of Fe_3_O_4_ Nanorods

A mixture of 220 mg of S2 and 10 g of OLA was prepared under N_2_ atmosphere and heated to 200 °C for 4 h. After that, the final black mixture was cooled down at room temperature and washed with acetone several times. After washing, the NPs were dried and stored as a solid. This sample was called S3.

#### 4.1.4. Synthesis of Mn-Doped Fe_3_O_4_ Nrs Using Fe_3_O_4_ Nanorods Seeds by a Cation-Exchange Protocol

A mixture of MnCl_2_ (13 mg, 0.1 mmol) in OLA (15 g) was heated at 120 °C for 20 min to completely dissolve the MnCl_2_. Next, 12 mg of S3 sample was dissolved in hexane, and then added to the solution under N_2_ atmosphere, and the reaction mixture was degassed under vacuum to remove the hexane. Then, TOP (1 mL) was injected under N_2_ atmosphere and the reaction mixture was heated to 200 °C for 30 min. The resulting brown-black mixture was cooled down to room temperature (RT) and acetone was added to precipitate the NPs. The solid was separated by centrifugation and washed with acetone several times until the supernatant was colorless, and the final black solid was dissolved and stored in hexane. This sample was named S3_Mn.

### 4.2. X-ray Diffraction Characterization

X-ray power diffraction (XRD) was performed at room temperature using a Bruker (Billerica, MA, USA.) D8 Twin, equipped with a monochromatic copper radiation source Cu K_α_ = 1.54 Å in the 25°–75° range with a step size of 0.05°/min.

### 4.3. Transmission Electron Microscopy Characterization

Conventional transmission electron microscopy (TEM) was performed on a FEI (Eindhoven, Netherlands) Tecnai Spirit microscope, operating at 120 kV and equipped with a lanthanum hexaboride thermionic electron source, a twin objective lens and a Gatan (Pleasanton, CA, USA) Orius CCD camera. High resolution TEM (HRTEM) imaging was performed on a double spherical aberration (Cs) corrected FEI Titan Themis microscope, operating at 300 kV and equipped with an ultrabright Schottky (XFEG) electron source and a FEI Ceta complementary metal oxide semiconductor (CMOS) camera. Finally, the scanning transmission electron microscopy (STEM) in high angular annular geometry (HAADF), along with energy-dispersive X-ray spectroscopy (EDS) elemental mapping, was carried out on a FEI Talos microscope operating at an acceleration voltage of 200 kV, also equipped with an XFEG electron source, a SuperX EDS equipped with a four SDD detectors spectrometer with an ultimate solid angle of X-rays collection 0.7 srad, and a FEI Ceta CMOS camera. The composition of the NRs was then determined by the Cliff–Lorimer method and always averaged over the whole nanoparticle volume. Size analysis was performed by measuring the length and width of clearly separated nanospindles and NRs. For each sample, 200 nanoparticles were measured, taking good care of exceeding the point when the length and width values became steady against additional measurements. Structural characterization was performed via analyzing the 2-Dimensional Fast Fourier Transform (2D-FFT) numerical diffractograms obtained from the HRTEM imaging and by measuring the planar and angular relationships occurring between diffraction spots appearing in the numerical diffractograms.

### 4.4. Magnetic Behavior Characterization

Magnetic characterization was performed on a Quantum Design (San Diego, CA, USA) VSM SQUID magnetometer equipped with a superconducting magnet producing fields up to 70 kOe (7 T) and a helium Quantum Design Evercool liquefier. Zero-field-cooled (ZFC) and field-cooled (FC) static (DC) magnetization curves were collected in the 4 ÷ 300 K thermal range upon a constant magnetic field with strength of 50 Oe. The magnetic features of magnetite-based pristine nanoparticles and the effect of Fe-to-Mn CE were studied by DC magnetometry according to the above-mentioned ZFC-FC magnetizations and hysteresis loops. The former was adopted to study temperature-dependent variations in magnetization, while the latter studied the isothermal variation in magnetization under a high magnetic field (up to ±70 kOe) at 4 and 300 K, corresponding to the magnetically blocked and unblocked states. Hysteresis loops were also measured twice (R0 indicates the hysteresis recorded after cooling, R1 refers to the hysteresis recorded immediately after the first one) after cooling the samples from RT down to 4 K in strong magnetic field of 70 kOe (FC hysteresis), to highlight both core/shell coupling-related and superficial disorder effects. The temperature corresponding to the maximum of the ZFC curve and the minimum temperature of superposition between the ZFC and FC curves are indicated as blocking temperature (T_B_) and irreversibility temperature (T_IRR_), respectively, and represent the two main parameters of the ZFC-FC magnetization curves. The derivative d(M_ZFC_ − M_FC_)/dT offers an effective way to observe variations in the anisotropy energy barrier as a function of temperature and its peaks are indicated as T_MAX_ [31]. The mean remanence (M_R_ = (|M_R+_| + |M_R−_|)/2) and loop vertical shift (ΔM_R_ = |M_R+_| − |M_R−_|)/2) were calculated from the upper and lower remanence magnetization values M_R+_ and M_R−_, while the mean coercivity (H_C_ = (|H_C1_| + |H_C2_|)/2) and exchange bias coercivity (H_E_ = (|H_C1_| − |H_C2_|)/2) were calculated from the negative and positive coercive fields, respectively labeled H_C1_ and H_C2_. Saturation magnetization values (M_S_) were determined from the hysteresis loops by extrapolation of M values vs. 1/H for 1/H→0. The samples were prepared according to a two-step protocol in order to normalize their magnetic response by the effective mass of the magnetic phase. At first, the solutions were dried and the resulting compounds were measured by thermogravimetric analysis (TGA) to assess the percentage of magnetic phase effectively formed. The dried compounds were then embedded in teflon tape to prepare the final SQUID sample. All the components of the SQUID samples were weighed for mass normalization.

## 5. Conclusions

By using a multi-step synthesis combined with a post-synthetic CE approach, we obtained partially hollow core/multiple-shell magnetic NRs. The presence of nanopores in the magnetite cores translates to the formation of structurally and magnetically disordered internal surfaces that impact on the magnetic properties of the NRs when compared to the bulk. Furthermore, a dramatic magnetic hardening effect was further promoted by the addition of a thin ferrimagnetic manganese oxide shell, which coupled antiferromagnetically with the more internal magnetic phases of the nanorods. Thus, this combined approach clearly represents a further step towards the magnetic tuning of magnetic nanoparticles not only by exploiting their shape and composition, but also by taking advantage of the hollow inner pores.

## Figures and Tables

**Table 1:** Sample dimensions. The nanocrystallite sizes were calculated by the conventional TEM imaging.

Samples	Length (nm)	Width (nm)	Aspect Ratio
S1	99.2 ± 16.8	15.0 ± 2.7	6.6
S2	96.5 ± 9.8	16.2 ± 1.6	6.0
S3	60.7 ± 12.3	14.6 ± 2.5	4.2
S3_Mn	70.2 ± 12.1	17.8 ± 2.9	3.9

**Table 2:** Magnetic parameters for the ZFC-FC curves for samples S3 and S3_Mn, determined as reported in the Materials and Methods section.

Sample	T_B_ (K)	T_IRR_ (K)	T_MAX_ (K)
S3	>300	>300	19
S3_Mn	40; >300	>300	8; 39

**Table 3:** Magnetic parameters for the hysteresis loops recorded at 4 K and RT (room temperature) for samples S3 and S3_Mn, determined as reported in the Materials and Methods section: H_C_ (mean coercive field), H_E_ (mean exchange bias), M_S_ (saturation magnetization); M_R_ (mean remnant magnetization); ΔM_R_ (mean vertical shift of the hysteresis loop); M_R_/M_S_ (reduced remnant magnetization).

Sample	H_C_ (Oe)	H_E_ (Oe)	M_S_ (emu/g)	M_R_ (emu/g)	ΔM_R_ (emu/g)	M_R_/M_S_
**Hysteresis at 4 K**						
S3	581	24	31.2	7.9	0.1	0.25
S3_Mn	4202	102	4.8	1.6	0.1	0.34
**Hysteresis at 4 K FC**						
S3	498	26	30.7	9.9	0.7	0.32
S3_Mn	4339	797	5.4	1.8	0.2	0.34
**Hysteresis at RT**						
S3	179	0	26.1	4.3	0.0	0.16
S3_Mn	74	0	4.7	0.1	0.0	0.03
